# The impacts of Covid-19 on perinatal mental health – Part 1

**Published:** 2022-09

**Authors:** O Ibiwoye, JE Hill, G Thomson

**Affiliations:** Liverpool Women’s Hospital; University of Central Lancashire; University of Central Lancashire

## Introduction

The novel coronavirus 19 (COVID-19) was declared a global pandemic by the World Health Organization (WHO) in February 2020. ^(1)^ While the virus may cause negative effects on physical health, the need to understand the impact of COVID-19 on mental health, particularly in vulnerable populations has been reported. ^(2)^

Pregnancy and childbirth can increase the risk of emotional instability and vulnerability to poor mental health. ^(3)^ The evidence before the pandemic showed a higher prevalence of depression and anxiety disorders in the perinatal period. ^(4)^ Perinatal mental illness affects up to 1 in 5 new and expectant mothers ^(5)^ with associated increased risk of miscarriage, preterm delivery, low birth weight, and lower Apgar score at birth. ^(6–10)^ Psychological stress during pregnancy may cause changes in physical activity, nutrition, and sleep, affecting maternal mood and foetal development. ^(11)^ Children of mothers who experienced high stress during pregnancy are more likely to have cognitive and behavioural problems and are at higher risk for later mental health problems. ^(12, 13)^ Prenatal anxiety and depression are also associated with brain structure and function changes in infants and children. ^(12)^

Recent studies show the increasing psychological burden of the COVID-19 pandemic on pregnant and post-partum women. ^(5,6)^ Pregnant women may be further vulnerable to anxiety due to increased concern about the transmission of the virus to their baby, however, there is not enough evidence of this effect. ^(14)^ According to the MBBRACE-UK report on COVID-19 related perinatal mortality, ten women died in the UK with SARS-CoV-2 between 01/03/2020 and 31/05/2020; four of these women committed suicide. ^(15)^

Given the above highlighted risks on the mother and child, there is a need to understand the impact of COVID-19 on perinatal mental health. The current review by Tomfohr-Madsen et al. 2021 ^(16)^ analysed the worldwide prevalence of depression and anxiety in the perinatal period during the COVID-19 pandemic. The evidence from this review may help prioritise interventions to alleviate the maternal mental health burden from COVID-19 and for future crises.

### Aim of commentary

This commentary aims to critically appraise the methods used within the review by Tomfohr-Madsen et al. 2021 ^(16)^ and to expand upon the findings in the context of clinical practice.

## Methods

This protocol registered rapid review by Tomfohr-Madsen et al. 2021 searched multiple databases of PsycINFO, EMBASE, MEDLINE and the Cochrane Central Register of Controlled Trails from the date of inception to February 10th, 2021. Only studies that reported the prevalence of depression and anxiety symptoms and/or diagnosis measured by a validated tool or by a clinician in pregnant women (≥18 years) which took place during the COVID-19 pandemic were included. Title, abstract and full paper screening was undertaken by two reviewers. Data extraction was undertaken by a single reviewer and checked by a second reviewer. A brief assessment of quality of the included papers was undertaken using a modified version of the National Institute of Health Quality Assessment Tool for Observation Cohort and Cross-Sectional Studies. An estimated prevalence rate was calculated using a random-effects meta-analysis. The difference between studies were assessed using I^2^ statistic and assessment of the influence of a single study was assessed by taking it away and reassessing the effects.

Meta-analysis combines quantitative evidence to summarise a whole body of research on a similar question, e.g., prevalence rates of depression and anxiety in perinatal women. A key threat to the validity of the meta-analysis is publication bias. Publication bias occurs when significant or clinically favourable results are more likely to be published than studies with non-significant or unfavourable results. In the Tomfohr-Madsen review, two methods were used to assess publication bias - a funnel plot (a scatter plot of the effect estimates from individual studies against some measure of each study’s size or precision) and Egger’s test (commonly used to assess potential publication bias in a meta-analysis via funnel plot asymmetry). The moderator factors (factors that may influence the findings) of maternal age, gestational age, % minority group members, study quality, country of study and time of data collection within the pandemic were assessed using subgroup analyses and meta-regression (a statistical test that assess the relationships between the factors).

## Results

Forty-six papers from East Asia (36%), Europe (22%), North America (20%), West Asia (11%), South Asia (9%) and Europe/West Asia combined (2%) were included. The mean age of participants was 30.63 years, and the mean gestational age was 23.78 weeks. For all combined prevalence estimates across the included studies, there was substantial heterogeneity (i.e., there was substantial differences in prevalence estimates between studies). The overall prevalence for clinically significant prenatal depression and prenatal anxiety was 25.6% (95% CI: 21.8% to 29.9%) and 30.5% (95% CI: 22.6% to 39.8%) respectively. There was no evidence of publication bias for prenatal depression but there was indication of possible publication bias (with a statistically significant Egger test) for prenatal anxiety. However, this could be a false positive due to the substantial between-study heterogeneity. ^(17)^

There was no evidence that any of the factors examined were associated with a change in prevalence rates of clinically significant prenatal depression. For prenatal anxiety there was no evidence of association for maternal age, gestational age, % minority, and study quality. However, there was a statistically significant lower prevalence (p <0.01) between studies undertaken in East Asia (16% 95% CI: 11% to 0.23%) compared to North America (43%, 95% CI: 24% to 63%) and Europe (44%, 95% CI: 27% to 62%). There was no evidence of difference between East Asia and West Asia. This finding therefore suggests that women in East and West Asia were less likely to experience prenatal anxiety and depression when compared to women from North America. There was also a statistically significant association with time of data collection within the pandemic and rates of anxiety. The prevalence rates of clinically significant anxiety levels were found to be higher within studies undertaken later in the pandemic (p < 0.04).

## Commentary

Using the Joanna Briggs Institute for Critical Appraisal Tools for Systematic Reviews and Meta-analysis, ^(18)^ nine of the 11 domains assessed were judged to be satisfactory for this review. The two domains rated as unsatisfactory were: a) the criterion for critical appraisal to be conducted by two or more reviewers independently was not achieved; and b) the criterion for assessing studies was not met, as while it refers to how the quality of studies was assessed the quality of each individual study was not reported, which in turn impacts the overall quality of the review.

According to Rucker et al. (2008), if there is substantial between-study heterogeneity as was the case in this review, then a specific test to assess publication bias - the arcsine test including random effects – should be undertaken. ^(19)^ As the authors used Egger’s test, their claims of publication bias need to be treated with caution. However, overall, it was deemed that this systematic review provides an accurate and comprehensive summary of the results of the available studies that address the question of interest.

Prior to the pandemic, available evidence suggests the prevalence of perinatal depression ranged from 10-15%. ^(20–22)^ According to the current review by Tomfohr-Madsen et al. 2021, this prevalence was found to have increased substantially to 25.6% and suggests the presence of clinical depression to be about one in four pregnant women. This is similar to results in other studies that assessed the prevalence of depression in pregnant women during the COVID-19 pandemic. ^(23)^ Although Tomfohr-Madsen et al.’s review found no statistically significant relationship between perinatal depression and any of the moderating factors that were assessed, Lopez-Morales et al., ^(23)^ found a significant effect of time on depression. They found that studies collected later in the pandemic reported increased severity of symptoms of depression in pregnant women.

In the current review, the prevalence of anxiety was 30.5%, affecting nearly one in every three pregnant women. There was also a longitudinal relationship in that the prevalence of clinically significant anxiety was higher in studies undertaken later in the pandemic. Evidence to date also shows that studies undertaken later in the pandemic demonstrate increased levels of anxiety in pregnant women. ^(23)^ Therefore, with the available evidence, the mental health of pregnant women stands a risk of further deterioration as the pandemic continues.

Overall, the perinatal period remains a high risk for new-onset and worsening of depression, anxiety, and other mental health symptoms for women, especially during the COVID-19 pandemic. As the pandemic remains, changes to service provision, for instance, virtual assessments with limited face to face contact, may impact access to specialist perinatal mental health services. It is therefore imperative that clinicians are aware of the increased risks during this period to enhance prompt identification and case management.

## Implications for midwifery

The need to put in place measures to triage the processes for early recognition and referral of pregnant women with mental health concerns to specialist services cannot be overemphasised. This should be facilitated by midwives undertaking routine screening for depression, anxiety, and other mental health disorders within the antenatal care pathway. Suitable care for women could then be facilitated through multidisciplinary team collaboration involving the obstetrician, midwives, occupational therapist, psychologists, and specialist perinatal mental health services. These measures would facilitate early recognition and treatment, thereby mitigating the negative consequences of mental illness on the mother, child, and family. Additionally, training of midwives about the ongoing impact of the COVID-19 pandemic on perinatal mental health should be undertaken to increase awareness and to enable early recognition and prevention.

## Key Points

- The COVId-19 pandemic is associated with an increased prevalence of depression and anxiety in the perinatal period
- It is imperative to prioritise the processes for early recognition, referral, and treatment of pregnant women with mental illness
- There is a need for further research to identify potential protective and risk factors for mental illness in the perinatal period, especially during the pandemic
